# Seroprevalence and risk factors for rubella infection in pregnant women attending a tertiary hospital in Kano-Nigeria

**DOI:** 10.11604/pamj.2023.46.97.39433

**Published:** 2023-12-04

**Authors:** Safiya Usman Zahradeen, Ibrahim Danladi Muhammad, Natalia Adamou, Ayyuba Rabiu, Mustapha Ahmed Yusuf, Samaila Adavuruku Danjuma Shuaibu, Garba Ibrahim

**Affiliations:** 1 Department of Obstetrics and Gynaecology, Aminu Kano Teaching Hospital, Kano, Nigeria,; 2 Department of Obstetrics and Gynaecology, Bayero University, Kano, Nigeria

**Keywords:** Rubella, antenatal, seroprevalence

## Abstract

**Introduction:**

rubella is a leading cause of vaccine preventable birth defects especially in developing countries. Acquisition of infection with the rubella virus in early pregnancy exposes the fetus to a very high chance of developing congenital rubella syndrome. The neonate is born with multiple abnormalities with the triad of congenital cataract, deafness and cardiovascular abnormalities like ventricular septal defect or patent ductus arteriosus. Limited data exist on the seroprevalence of rubella antibodies in pregnant women in Nigeria. The aim of this study was to determine the seroprevalence of rubella antibodies in pregnant women attending antenatal clinic in Aminu Kano Teaching Hospital, Kano State.

**Methods:**

the study was a cross-sectional study involving one hundred and sixty-three pregnant women attending antenatal clinic of Aminu Kano Teaching Hospital in Kano, Nigeria. Interviewer administered questionnaire was used to collect sociodemographic data and risk factors. Blood samples were taken from consenting pregnant women during antenatal care and samples were subjected to antibody testing (IgG and IgM). Descriptive analysis was done for sociodemographic data and seroprevalence of rubella. Chi-square tests were used to determine associations.

**Results:**

one hundred and sixty-three pregnant women were recruited for the study. The participants´ age ranged from 18 to 41 years with mean age of 27.60±5.7 years. The overall rubella seroprevalence was found to be 68.7%. The seroprevalence of specific anti-Rubella virus IgM and IgG was found to be 58.4% and 37.3% respectively while prevalence of having both anti-Rubella virus IgG and IgM in the women was found to be 26.4%. Non-formal education and immunodeficiency was found to be associated with rubella infection (P-value of 0.018 and 0.001 respectively).

**Conclusion:**

the study found a high prevalence of anti-Rubella virus immunoglobulins in asymptomatic pregnant women attending antenatal care in our facility with immunodeficiency and non-formal education found to be significant risk factors.

## Introduction

Rubella is a contagious disease caused by the Rubella virus [1]. It is also called ‘German measles’ or three-day measles. Rubella virus is an enveloped single stranded RNA virus belonging to the family *Togaviridae*, genus *Rubivirus* [2,3]. Rubella virus infection is transmitted by respiratory droplets and causes generally mild and self-limiting symptoms including low-grade fever, malaise, arthralgia, lymphadenopathy, upper respiratory symptoms, sore throat, maculopapular rash [2,3]. The virus grows and replicates in the nasopharynx, followed by multiplication in the cervical lymph nodes, then enters the blood stream and disseminates. It has an incubation period of 2-3 weeks and humans are the only known host of the rubella virus [1]. Infection with Rubella virus during early stage of pregnancy may cause fetal demise or lead to congenital rubella syndrome (CRS) in the infant. Congenital Rubella infection usually occurs when the mother gets the virus within the first trimester of pregnancy or just before conception [4]. The neonate is born with congenital birth defects involving many organs which include which involves the eyes, ears, heart, brain and the endocrine system causing deafness, mental retardation, ocular manifestation (as loss of sight, cataract, glaucoma, retinitis, microphthalmia), patent ductus arteriosus, VSD, microcephaly, late onset diabetes mellitus and thyroid disorder like hyperthyroidism amongst others [5,6]. Rubella is a leading cause of vaccine preventable birth defects especially in developing countries and is one of the known causes of autism [7]. Congenital rubella syndrome is globally a public health concern with more than 100,000 cases reported annually worldwide in the newborns [5]. Before the rubella vaccine was first introduced and licensed in the United States in 1969, rubella was a common disease that occurred primarily among young children. However, the introduction of rubella vaccines led to its eradication from the United States in 2004. Since then, less than 10 cases have been reported annually with most cases were found in immigrants that entered the country [7,8]. However, Rubella remains endemic in many other countries especially in sub-Saharan Africa [9,10]. The World Health Organization (WHO) recorded that rubella cases in the African Region and the South-East Asian Region alone increased from 865 to 17,388 and from 1,165 to 17,208 from 2000 to 2009, respectively. These regions represent a significant number of the 121,344 global cases of rubella reported by the WHO during 2009. Unfortunately, none of these regions have any clear or specific goals to overcome the rubella outbreaks [5].

The prevalence of Rubella infection in children is equal in both sexes whereas it affects more adult females than males [11]. In the pre-vaccine era, it affects children more than adults but currently adults more than 20 years are more affected. Risk factors for Rubella infection include partially or unvaccinated individual, travel to endemic areas, exposure to household members with Rubella and immunodeficiency [11]. About 25% - 50% of those exposed with acquired rubella infection may be asymptomatic or subclinical, especially in children [12]. In those who clinically manifest the disease, their symptoms are mild and self-limiting. There is a prodromal period of one to five days after the infection and is represented by a low-grade fever, nausea, anorexia, lethargy, coryza, cough, headache, non-exudative conjunctivitis, malaise, lymphadenopathy and an upper respiratory infection [12].

Laboratory confirmation of rubella virus infection can be based on a positive serological test for rubella-specific immunoglobulin M antibody, a four-fold or greater increase in rubella-specific immunoglobulin G titres between acute and convalescent sera or detection of rubella virus RNA by reverse transcriptase-polymerase chain reaction and also viral culture from body fluids [11]. The haemaglutinin inhibition test was once the standard test for antibodies to rubella virus and many tests are calibrated using the haemaglutinin assay [13]. The most widely available test is the ELISA to detect the viral antibody IgM and IgG [14,15]. ELISA is commonly used because it is sensitive, highly specific, technically easy to perform, rapid and relatively not expensive. Specimens for the diagnosis of rubella by virus culture usually consist of throat swabs (TS), oral fluids (OF) or nasopharyngeal secretions, and by antibody detection are usually sera or oral fluid [11,15]. The rubella virus IgM appears soon after acute infection but later disappears, it indicates acute infection but is generally undetected after 4 to 6 weeks after rash onset [11]. False positive results usually occur in presence of rheumatoid factor, infection with parvovirus, CMV infection or a positive heterophile test for infectious mononucleosis [13]. The use of IgM capture ELISA rather than Indirect IgM ELISA may reduce the occurrence of false positive results. IgG antibodies usually persist for life. The aim of this study was to determine the seroprevalence and risk factors for rubella infection in pregnant women attending antenatal clinics in Aminu Kano Teaching Hospital, Kano State. The study objectives were to determine the prevalence and risk factors of rubella infection among pregnant women attending antenatal care at Aminu Kano Teaching Hospital, Kano.

## Methods

**Study design:** the study was a cross-sectional descriptive study.

**Study setting:** the study was conducted at the departments of Obstetrics/Gynaecology and Microbiology of Aminu Kano Teaching Hospital, Kano-Nigeria.

**Participants:** the subjects for this study were pooled out from the population of pregnant women from 38 to 40 weeks of gestation attending the antenatal care clinic of Aminu Kano Teaching Hospital. All consenting pregnant women who presented for follow-up visits were included in the study population. Women who received rubella vaccines and those who earlier tested positive for rubella antibodies in their lifetime were excluded from the study. Patients with rheumatoid arthritis were also excluded.

**Variables:** sociodemographic variables include age, marital status, occupation, educational status and place or residence. Rubella seroprevalence were interpreted as negative for IgG or IgM antibody to rubella virus positive for IgG or IgM antibody to rubella virus.

### Data sources/measurement

A structured interviewer administered proforma was administered to the study participants after obtaining written informed consent. The information obtained includes the sociodemographic data, risk factors associated with rubella virus infection, area of residence and immunodeficiency (HIV, diabetes mellitus and asthmatic patients). Five (5) mls of venous blood was obtained and transferred into an EDTA bottle. The sample bottles were coded with the date taken and according to the numbers assigned to each patient which corresponded to the patient proforma. The samples were taken to the microbiology laboratory immediately for processing, preservation and analysis. The samples were centrifuged at 2500 rpm for 10 min to allow the sera to be separated. The sera was collected using clean Pasteur pipettes, transferred into serum containers, and stored in a refrigerator set at 2-8°C until analysed.

The screening test for the rubella antibodies (IgG and IgM) was done after pooling the maternal samples using ELISA. Purified rubella antigen was coated on the surface of micro wells. Diluted patient serum was added to wells and the rubella IgG specific antibody, if present, bound to the antigen. All unbound materials were washed away. After adding enzyme conjugate, it bound to the antibody-antigen complex. Excess enzyme conjugate was washed off and TMB chromogenic substrate was added. The enzyme conjugate catalytic reaction was stopped at a specific time. The intensity of the color generated was proportional to the amount of IgG specific antibody in the sample. The results were read by a microwell reader compared in a parallel manner with calibrator and controls. IgM qualitative test was also carried out on the samples using a similar method as the Rubella IgG qualitative test. Test results were interpreted as follows: Negative-Rubella G index less than 0.90 are negative for IgG or IgM antibody to rubella virus; Positive - Rubella G index of 1.00 or greater are positive for IgG or IgM antibody to rubella virus.

**Bias:** all laboratory analysis was conducted by same individual to minimise inter-observer bias.

**Study size:** the minimum sample size was obtained using the fisher´s formula. Using prevalence of 90.2% from similar previous study done at Zaria [2]. A total of one hundred and sixty-three patients were recruited for the study using a systematic sampling technique. Systematic sampling method was used to recruit patients. In the ante-natal clinic an average of 20 patients presented for follow-up per day, making up to 600 follow-ups monthly. Hence the sampling frame was calculated to be 4. The first patient recruited among the consenting patients that met the inclusion criteria was by simple balloting, there after every fourth patient was recruited until the sample size was achieved. Patient recruitment was commenced in May 2022 and conducted over a period of 8 weeks with about 20 patients recruited per week.

**Statistical methods:** data obtained in the proforma was analyzed using the statistical package for social science SPSS version 25 (SPSS inc, Chicago, IL USA, 2015). Descriptive data were expressed as frequencies and percentages for qualitative data while the quantitative data was expressed in mean and standard deviation. Chi-square test were used to test for statistical significance. Ethical approval for the study was obtained from the research and ethics committee of Aminu Kano Teaching Hospital, Kano.

**Ethical considerations:** ethical approval for the study was obtained from the research and ethics committee of Aminu Kano Teaching Hospital, Kano. Ref No AKTH/MAC/SUB/12A/P-3/VI/3385(27th April 2022). The study was explained to the participants/clients in details, informed written consent was sought from the patients who indicated interest to participate in the study, there by recruiting them into the study. Confidentiality was assured and they were free to withdraw from the study at any point if they wished to without denying them health care services. Cost of the tests was sponsored by the researchers.

## Results

**Participants:** the total number of study participants that completed the study and their samples analysed were one hundred and sixty-three.

### Descriptive data

The age of the participants ranged between 18 to 41 years with mean age of 27.60±5.7 years. Women aged 20 - 29 years were predominant constituting about 59.5% of the participants. The least were those aged 40 years and above accounting for 2.5% of the women. All the women were married and majority of the participants were Muslims from Hausa tribe. Majority (70.6%) of the women were not gainfully employed. A significant majority of them (93.3%) had formal education and 87.7% reside in an urban area in the state. This is as shown in Table 1.

**Table 1:** sociodemographic and reproductive characteristics

Variables	Frequency (n=163)	Percentage (%)
**Age**		
< 20	6	3.6
20-29	97	59.5
30-39	56	34.4
>40	4	2.5
**Marital status**		
Married	163	100
Single	0	0
Religion		
Muslim	156	95.7
Christian	7	4.3
**Occupation**		
Unemployed	115	70.6
Self Employed	32	19.6
Civil servant	16	9.8
**Education**		
Formal	152	93.3
Non-formal	11	6.7
Ethnicity		
Native (Hausa)	129	79.1
Non- Hausa (Others)	34	20.9
**Area of residence**		
Urban	143	87.7
Rural	20	12.3

**Outcome data/main results:** the overall seroprevalence of rubella was found to be 68.7%. The prevalence of anti-Rubella virus IgG as found to be 37.4% while that of anti-Rubella IgM was found to be 58.3%. The prevalence of having both anti-Rubella virus IgG and IgM was 26.4%. This is as shown in Table 2.

**Table 2:** prevalence of rubella antibodies among pregnant women

Test	Positive Freq (%)	Negative Freq (%)	95% CI (%)	Total
IgM	95(58.3)	68(41.7)	50.3-65.8	163
IgG	61(37.4)	102(62.6)	30.0-45.3	163
IgG or IgM	112(68.7)	51(31.3)	61.0-75.5	163
IgG & IgM	43(26.4)	120(73.6)	20.0-33.8	163

### Other analyses

Non-formal educational level and immunodeficiency were found to be statistically significantly associated with presence of anti-rubella immunoglobulins with P-values of 0.018 and 0.001 respectively. However, there was no statistically significant association between presence of antibodies with age, religion, occupation, ethnicity, area of residence, husband´s occupation and exposure to children with rubella infection. This is shown in Table 3.

**Table 3:** risk factors associated with rubella infection in pregnant women

Variables	Rubella Seropositive N=112	Rubella Seronegative N=51	P-Value
**Age**			0.781
< 25	40(71.4)	16(28.6)	
25-35	62(68.1)	29(31.9)	
≥35	10(62.5)	6(37.5)	
**Religion**			1.00
Islam	107(68.6)	49(31.4)	
Christianity	5(71.4)	2(28.6)	
**Occupation**			0.745
Unemployed	81(70.4)	34(29.6)	
Self Employed	21(65.6)	11(34.4)	
Civil servant	10(62.5)	6(37.5)	
**Education**			0.018*
Non-formal	11(100)	0(0)	
Formal	101(66.4)	51(33.6)	
**Ethnicity**			0.306
Hausa	86(66.7)	43(33.3)	
Non-Hausa	26(76.5)	8(23.5)	
**Husband's occupation**			0.174
Self Employed	62(64.6)	34(35.4)	
Civil servant	50(74.6)	17(25.4)	
**Place of Residence**			0.900
Urban	98(87.5)	45(88.24)	
Rural	14(12.5)	6((11.76)	
**Immunodeficiency**			0.001*
Present	21(18.75)	0(0)	
Absent	91(81.25)	51(100)	
**Exposure to children**			0.800
Exposed	55(49.11)	26(50.98)	
Unexposed	57(50.89)	25(49.02)	

*Statistically significant

## Discussion

### Key findings

This study was conducted to determine the burden of rubella infection in our community to make justification for the inclusion of MMR vaccine in the national immunization program in Nigeria. The study found the overall seroprevalence of rubella to be 68.7% of antenatal clients. The prevalence of anti-Rubella virus IgG as found to be 37.4% while that of anti-Rubella IgM was found to be 58.3%. The prevalence of having both anti-Rubella virus IgG and IgM was 26.4%.

### Interpretation

The overall seroprevalence of rubella in this study was found to be high. Similar findings have been reported in Ibadan, Nigeria (68%) [16], DRC (58.9%) [17] and Sudan (65%) [18]. However, higher prevalences were reported by previous studies in Ethiopia (89%) [19], Senegal (90.1%) [20], Namibia (85.0%) [21], Burkina Faso (95%) [22] and Zimbabwe (92%) [23]. The variation in prevalence with these studies might be due to difference in sample sizes, disease endemicity, and differences in the cut-off points of the assays.

The seroprevalence of rubella specific IgG in the pregnant women in this study was found to be 37.4%. The positivity of IgG indicates evidence of chronic infection. The pregnant women could have been exposed to the virus before pregnancy or during the first trimester. Exposure to the virus in the first trimester might cause the congenital rubella syndrome (a triad of microcephaly, deafness and congenital cataract). However, it was much lower than the figures of 97.2% and 90.2% reported from Zaria in 2010 [24] and 2013 [2] respectively. Higher prevalence of 79.5% was also reported in Ethiopia [19]. The higher prevalence of rubella antibodies in those studies when compared to this study could be due to higher sample size in the studies. The prevalence of IgM to be 58.4% in this study signifies evidence of recent infection. This study was done in the third trimester as affectation of the neonate with congenital rubella syndrome is decreased compared to infection with the virus in the first trimester. It is evidence that rubella infection is still ongoing in the populace. The high prevalence of IgM could be due to the timing of the study (done in the hot dry season), in which so many studies showed increase in rubella infection between the first quarter of the year. A study done in kebbi [4] showed increase in rubella infection in the month of march. This is also similar to a study done in south western Nigeria [25] which showed high prevalence of rubella infection between January to April. Comparing this study with those done in Ibadan [26], Ethiopia [19] and Benin [27], much lower prevalences of 1.84%, 9.5% and 10% were reported respectively for rubella specific IgM. Immunodeficiency like HIV, diabetes mellitus and steroid intake (asthmatic patients) being non-modifiable factors, increases the susceptibility to Rubella infection in women. Significant association was reported between women that were immune-deficient and the presence of rubella infection. Children have depressed immunity thereby they are susceptible to rubella. Pregnant women when in contact to these children as their teachers or a house with so many children can contact the rubella infection. However, in this study there was no association between exposure to children and rubella infection.

### Generalisability

The high seroprevalence of Rubella in this study indicate a high prevalence in our environment which might result from lack of childhood immunization to the virus as MMR vaccine is not included in the national immunization program in Nigeria. Rubella infection is usually asymptomatic and the presence of symptoms in a patient is hardly regarded as rubella infection until with high index of suspicion because its symptoms are close to that of common cold and measles.

## Conclusion

This study found a high prevalence of rubella infection in women attending antenatal care in Kano with seroprevalence of anti-RV IgM higher than IgG. Immune and educational statuses of the women were found to be the significant risk factors for rubella seropositivity.

### What is known about this topic

- *Rubella is endemic in many countries in sub-Saharan Africa including Nigeria;*
- *Majority of acquired rubella infections may be asymptomatic or subclinical*.

### What this study adds

- *This study demonstrated a high prevalence of rubella infection among pregnant women;*
- *This will justify the inclusion of MMR vaccine in the national immunization program in Nigeria;*
- *Immunodeficiency increases susceptibility to rubella infection*.
